# Crab shell polypeptides enhance calcium dynamics and osteogenic activity in osteoporosis

**DOI:** 10.3389/fphar.2025.1605422

**Published:** 2025-08-25

**Authors:** Xiaolei Dong, Guangmin Zhang, Chong Sun, Hui Zhang, Jinmeng Zhen, Xinlei Li, Xiaohui Xu, Jiane Liu, Xiangzhong Zhao, Yiming Zhang, Linlin Liu, Shaoqi Tian, Daijie Wang, Zheng Wang, Bing Li

**Affiliations:** ^1^Department of Genetics and Cell Biology, School of Basic Medicine, Qingdao University, Qingdao, Shandong, China; ^2^Department of Reproductive Medicine, The Affiliated Hospital of Qingdao University, Qingdao, Shandong, China; ^3^Department of Spinal Surgery, The Affiliated Hospital of Qingdao University, Qingdao, Shandong, China; ^4^Department of Medicinal Chemistry, School of Pharmacy, Shandong University of Traditional Chinese Medicine, Jinan, Shandong, China; ^5^Department of Pharmaceutical Analysis, School of Pharmacy, Shandong University of Traditional Chinese Medicine, Jinan, Shandong, China; ^6^Medical Research Center, The Affiliated Hospital of Qingdao University, Qingdao, Shandong, China; ^7^Department of Orthopedic Operation, The Affiliated Hospital of Qingdao University, Qingdao, Shandong, China; ^8^International Joint Laboratory of Medicinal Food R&D and Health Products Creation/Biological Engineering Technology Innovation Center of Shandong Province, Heze Branch of Qilu University of Technology (Shandong Academy of Sciences), Heze, Shandong, China

**Keywords:** crab shell polypeptides, osteoporosis, calcium dynamics, osteogenic activity, OP treatment

## Abstract

**Background:**

Osteoporosis (OP) is a chronic, systemic skeletal disorder characterized by progressive bone loss and microarchitectural deterioration, which increases fracture susceptibility and presents a challenging set of global healthcare problems. Current pharmacological interventions are limited by adverse effects, high costs, and insufficient long-term efficacy. Here, we identify snow crab shell-derived polypeptides (SCSP) as a potent osteoprotective agent.

**Methods:**

SCSP were extracted and characterized. Using an ovariectomized (OVX) mouse osteoporosis model, mice received daily oral SCSP (50, 100 mg/kg) or saline for 8 weeks. Bone microstructure (micro-CT), histomorphometry (H&E, Masson, TRAP), immunohistochemistry, and serum bone turnover markers were analyzed. In vitro, SCSP (100, 200 μg/ml) effects on osteogenic/adipogenic differentiation in MSCs/preosteoblasts were assessed via staining (ARS, ALP, Oil Red O) and molecular analyses (Western blot, qPCR, RNA-Seq).

**Results:**

SCSP, enriched in glutamic acid, aspartic acid, and lysine, significantly enhances bone mineral density, restores trabecular architecture, and preserves bone tissue integrity in an ovariectomy-induced OP mouse model without detectable systemic toxicity. At the molecular level, SCSP treatment induces the expression cell cycle regulators and motor protein pathways in osteoblasts while suppressing pro-inflammatory signaling networks, thereby re-establishing osteoblast-osteoclast balance and restoring calcium and phosphorus homeostasis. This combined mechanism promotes osteogenesis while simultaneously suppressing adipogenesis.

**Conclusion:**

Our findings position SCSP as a promising natural therapeutic for OP and provide key mechanistic insights that may guide future bone-targeted interventions.

## Introduction

Osteoporosis (OP) is a systemic skeletal disorder characterized by decreased bone mass, deterioration of bone microarchitecture, and an increased risk of fragility fractures (Zhang et al., 2022; Tang et al., 2024). The global number of OP cases between 2030 and 2034 is estimated to increase to 263.2 million, exacerbating the global healthcare burden (Zhu et al., 2023). The prevalence of OP is significantly higher in females than in males, particularly among postmenopausal women, due to estrogen deficiency-induced bone resorption (Alswat, 2017). The pathophysiology of OP is primarily driven by an imbalance between bone resorption and bone formation, resulting from reduced osteoblast (OB) proliferation and differentiation (Pan et al., 2018), excessive osteoclasts (OC) activation (Chen et al., 2024), and dysregulated calcium metabolism (Wang et al., 2024). Current pharmacological interventions for OP include anti-resorptive agents, such as bisphosphonates (Kuźnik et al., 2020), and denosumab, anabolic agents, such as parathyroid hormone analogs (Leder, 2017), and supportive treatments like active vitamin D and calcium supplements (Capozzi et al., 2020). Notwithstanding their clinical benefits, these therapies fail to address the underlying disease mechanisms and are often associated with significant adverse side effects, high costs, and limited long-term efficacy.

Crustacean shells are rich in polysaccharides, proteins, lipids, and minerals such as calcium, phosphorus, and magnesium, as well as compounds including astaxanthin and β-carotene. These components exhibit unique bioactive properties, biocompatibility, and low toxicity (Vilasoa-Martínez et al., 2008; Crespo et al., 2006; Beaulieu et al., 2009; Taksima et al., 2019). For example, chitin, a key polysaccharide, exhibits potent anti-inflammatory, antioxidant, antimicrobial, wound-healing (Chotphruethipong et al., 2023; literature review of pathology et al., 2014), and anti-tumor capabilities (Younes et al., 2014). Similarly, lipids derived from crustacean shells have demonstrated anti-inflammatory and neuroprotetive properties (Tsoupras et al., 2024; Cholidis et al., 2024; Abraúl et al., 2023). Astaxanthin is recognized for its antioxidant, anti-inflammatory, and skin-protective and anti-skin carcinogenesis properties (Chintong et al., 2019; Rao et al., 2013), while β-Carotene contributes to antioxidant defense, vision support, and immune modulation (Jiang et al., 2024; Rammuni et al., 2019; Nair et al., 2023). Nevertheless, the bioactivity and therapeutic potential of crustacean shell-derived proteins remain largely unexplored. The process of calcium deposition in crustaceans is a highly regulated biomineralization process, and matrix proteins within the shell play critical roles in nucleation, stabilization, and orchestrated calcium deposition, thereby contributing to the mechanical strength of the exoskeleton (Nagasawa, 2012; Nagasawa, 2011; Abehsera et al., 2018; Shaked et al., 2024). To this end, we hypothesize that proteins derived from crustacean shells may play a pivotal role in regulating calcium homeostasis in bone tissue.

Here, we extract and characterize the enzymatically hydrolyzed peptides from snow crab shells, and demonstrate that these snow crab shell derived polypeptides (SCSP) exhibit potent anti-osteoporotic activity in a bilateral ovariectomy-induced osteoporosis mouse model. Mechanistically, SCSP enhances calcium deposition, promotes OB activity, and inhibits OC function. Based on chemical, biochemical, bioinformatics, and functional data detailed below, we propose SCSP as a promising natural candidate for improving bone health and provide new insights and therapeutic strategies for OP treatment.

## Materials and methods

### Reagents and antibodies

Antibody against RUNX-2 and COL-1 were purchased from Cell Signaling Technology. Antibodies against OSX, NFATc1, RANKL, and CTSK were purchased from Santa Cruz Biotechnology. Antibodies against BMP-2 was purchased from Servicebio (Table 1). Hematoxylin and Eosin (H&E) Staining Kit (C0105S) and BCIP/NBT Alkaline Phosphatase (ALP) Color Development Kit (C3206) were purchased from Beyotime. Masson’s trichrome staining solution (G1006) and tartrate-resistant acid phosphatase (TRAP) staining reagents (G1050) were purchased from Servicebio. Alizarin Red S (ARS) solution (G1452) was purchased from Solarbio, and Oil Red O staining solution (320-06-5) was purchased from Sigma-Aldrich.

**TABLE 1:** Antibodies used for Western blot (WB) and Immunohistochemistry (IHC).

Antibody	Catalog number	Dilution (WB)	Dilution (IHC)	Vendor
RUNX-2	#12556	1:1000	1:100	Cell Signaling Technology
OSX	sc-393325	1:1000	—	Santa Cruz Biotechnology
NFATc1	sc-7294	1:1000	—	Santa Cruz Biotechnology
RANKL	sc-377079	1:1000	—	Santa Cruz Biotechnology
CTSK	sc-48353	1:1000	—	Santa Cruz Biotechnology
GAPDH	E-AB-48016	1:1000	—	Santa Cruz Biotechnology
BMP-2	GB11252	—	1:100	Servicebio
COL-1	#72026	—	1:100	Cell Signaling Technology

### Extraction and characterization of SCSP

Alaskan snow crab (Genus: *Chionoecetes,* Species: *Opilio)* caught wild in the United States. Fresh snow crab shells were cut into ∼1 cm pieces, crushed into fragments, washed, pH-adjusted to 10 with 0.2 mol/L KOH, and hydrolyzed at 70°C under constant stirring for 3 h. The hydrolysate was filtered, neutralized with acetic acid, hydrolyzed using papain for 4 h at 37°C, vacuum-concentrated, precipitated using ethanol, and vacuum-dried at 40°C. The molecular weights of SCSP were determined based on viscosity and retention time using a PL aquagel-OH Mixed-H Column (8 μm, 7.5 × 300 mm, Agilent) coupled with a Refractive Index Detector (Agilent) and Multi-Angle Laser Light Scattering Detector (Agilent) at 45°C. Amino acid composition was analyzed by hydrolyzing samples in 6M hydrochloric acid at 110°C for 22 h, followed by chromatography with Sulfonic Acid Cation Exchange Resin Columns (Agilent). Detections were performed at wavelengths of 570 nm and 440 nm.

### Experimental animals

Five-week-old female C57BL/6 mice (20 ± 5 g) were purchased from Home-SPF Biotechnology Co., Ltd. (Beijing, China). Mice were housed in an SPF-grade facility at Qingdao University under controlled conditions (25°C ± 3°C, 60%–70% humidity, 12-h light/dark cycle). All animal procedures followed ethical guidelines approved by the Shandong Provincial Laboratory Animal Management Committee and the Experimental Animal Center of Qingdao University (QDU-AEC-2024418).

### Ovariectomy (OVX) mice model

Mice were anesthetized, and the surgical area was shaved and sterilized with iodine. The skin, mucosa, and muscle layers were incised sequentially, and a dorsal incision was made approximately 2 cm lateral to the spine at the level of the last rib. Both ovaries were ligated at the oviduct and excised. A total of 24 female C57BL/6 mice were randomly assigned into 4 groups: Sham-operated (Sham), osteoporotic model (OVX), OVX+50 mg/kg SCSP treatment (Low-SCSP), and OVX+100 mg/kg SCSP treatment (High-SCSP). The SCSP treatment groups received SCSP via oral gavage daily, while the Sham and OVX groups received equivalent volumes of saline. All treatments were continued for 8 weeks before femur collection.

### Micro-CT scanning

Femurs were fixed in 4% paraformaldehyde (PFA) and scanned using a Micro-CT System (Quantum GX2, PerkinElmer, Japan) at 90 kV and 200 μA. 100 layers at the proximal end of the tibial platform were selected for statistical analysis of cortical layer thickness, trabecular structure, and bone marrow cavity volume using Analyzer 12.0 Software (PerkinElmer). The volume of interest (VOI) was positioned at the proximal tibial metaphysis, starting precisely 0.5 mm distal to the growth plate to exclude the primary spongiosa and epiphyseal tissue. 100 layers = 1 mm: 100 layers × 10 μm = 1000 μm (1 mm).

### Histological staining

Femurs were decalcified, embedded in paraffin, and sectioned for histological analysis. For H&E staining, sections were deparaffinized, stained with hematoxylin for 2 min and eosin for 10 s, washed with water, and imaged. For masson trichrome staining, sections were stained with Weigert’s iron hematoxylin for 10 min, sequential stained with acid ethanol, masson blue, aniline blue, and imaged. For TRAP staining, sections were incubated with TRAP solution at 37°C for 30 min in the dark, counterstained with hematoxylin, and imaged. All the histological staining were imaged with a light microscope (Ni-U, Nikon, USA).

### Immunohistochemistry (IHC)

Decalcified bone sections were dewaxed, rehydrated, quenched, antigen-retrievaled, blocked with 5% BSA, incubated overnight at 4°C with primary antibodies, washed, incubated with secondary antibodies, counterstained with hematoxylin, and imaged with a light microscope (Nikon, USA).

### Serum biochemical analysis

Urine was collected and centrifuged at 13,000 rpm for 5 min to obtain the supernatant. Blood was collected via orbital puncture and centrifuged at 3,000 rpm for 10 min. The concentrations of calcium (Ca^2+^) and inorganic phosphate (Pi) in serum and urine were measured using Calcium (Ca^2+^) colorimetric Assay Kit (E-BC-K103-M, Elabscience) and Phosphorus (Pi) Colorimetric Assay Kit (E-BC-K245-M, Elabscience) according to the manufacturer’s instructions. Absorbance were recorded at 610 nm (Ca^2+^) and 660 nm (Pi) using a microplate reader (SpectraMax iD3, Molecular Devices, USA).

### Cell culture and differentiation

Bone marrow mesenchymal stem cells (MSCs) (CP-M131) was purchased from Pricella. MSCs were cultured in BC-T4 medium (04304P05, Baso) supplemented with 10% FBS (A5670701, Gibco). Murine MC3T3-E1 preosteoblasts were cultured in α-MEM media supplemented with 10% FBS. Cultures were passaged every 3-4 days by adding 0.25% trypsin (25300054, Gibco) for 5-10 min and re-plating at a 1:4 ratio. Osteogenic differentiation was induced with DMEM medium containing 10 mM β-glycerophosphate (HY-126304, MCE), 100 nM dexamethasone (HY-14648, MCE), and 50 μM L-ascorbic acid (HY-B0166 MCE) for 21 days (Choi et al., 2009). Adipogenic differentiation was induced with DMEM containing 100 μg/mL 3-isobutyl-1-methylxanthine, 1 μM dexamethasone (HY-14648, MCE), and 50 μg/mL ascorbic acid for 12 days (Liu et al., 2023). All cultures were maintained at 37 °C and 5% CO_2_. Cell experiments were divided into 4 groups: untreated group (Normal), differentiated group (Control), 100 μg/mL SCSP group (Low-SCSP) and 200 μg/mL SCSP group (High-SCSP).

### Cell viability assay

5 × 10^3^ cells per well were seeded in a 96-well plate and cultured in complete medium for 24 h. After SCSP treatment for 48 h, 10 μL of CCK-8 (Yeasen) solution was added to each well, and incubated for 2 h at 37°C in the dark. Absorbance was measured at 450 nm using a full-wavelength microplate reader (SpectraMax iD3, Molecular Devices, USA).

### Cell staining analysis

Cells were fixed with 4% PFA for 20 min and washed, stained with 1% ARS solution, BCIP/NBT staining solution, or Oil Red O solution, incubated at room temperature in the dark for 30 min, washed, and imaged with a light microscope (Ni-U, Nikon, USA).

### Western blot analysis

Cells or tissues were lysed with RIPA buffer containing protease and phosphatase inhibitors (E-BC-R327, Elabscience), homogenized, and incubated on ice for 10 min. The supernatant protein concentration was determined using a BCA assay. Protein lysate was resolved on SDS-PAGE gel and transferred onto a PVDF membrane (1620177, BIO-RED). Blots were blocked in 5% non-fat dry milk, incubated with primary antibody overnight at 4°C, washed, incubated with secondary antibody for 1 h at room temperature, washed, and developed with Super Excellent Chemiluminescent Substrate Detection Kit (E-IR-R308, Elabscience).

### RNA isolation and qPCR

Total RNA was isolated using FreeZol Reagent (R711-01, Vazyme), precipitated, washed with 70% ethanol and dissolved in H_2_O. 1 μg of total RNA was reverse transcribed using random hexamers and Hiscript III Reverse Transcriptase (R302-01, Vazyme). 20 ng cDNA was used in each RT-qPCR reaction on a CFX96 instrument using Taq Pro Universal SYBR qPCR Master Mix (Q712-02, Vazyme). The primers used for qPCR were listed in Table 2.

**TABLE 2:** Primer sequences used for qPCR analysis.

Gene name	Forward primer (5'→3′)	Reverse primer (5'→3′)
*Runx-2*	ATG​CTT​CAT​TCG​CCT​CAC​AAA	GCA​CTC​ACT​GAC​TCG​GTT​GG
*Bmp2*	GGG​ACC​CGC​TGT​CTT​CTA​GT	TCA​ACT​CAA​ATT​CGC​TGA​GGA​C
*Opg*	ACC​CAG​AAA​CTG​GTC​ATC​AGC	CTG​CAA​TAC​ACA​CAC​TCA​TCA​CT
*Rankl*	CAG​CAT​CGC​TCT​GTT​CCT​GTA	CTG​CGT​TTT​CAT​GGA​GTC​TCA
*Gapdh*	TCC​CAC​TCT​TCC​ACC​TTC​GAT​GC	GGG​TCT​GGG​ATG​GAA​ATT​GTG​AGG

### RNA sequencing (RNA-Seq) analysis

RNA libraries were prepared using the VAHTS^®^ Universal V8 RNA-Seq Library Prep Kit (NRM605, Vazyme). Sequencing was performed on the MGI-SEQ 2000 platform. Reads were aligned to the mouse genome (GRCm38) using HISAT2, and differential expression analysis was conducted using DESeq2. GO and KEGG pathway enrichment analyses were performed using WebGestalt.

### Statistical analysis

All the data conform to a normal distribution. Data were represented as means ± SEM. Statistical comparisons were performed using GraphPad Prism 9.5, employing one-way ANOVA, two-way ANOVA, or Student’s t-test. Significance was defined as *P* < 0.05.

## Results

### Characterization of SCSP

To characterize SCSP, we first quantified its yield following enzymatic hydrolysis. After 4 h of hydrolysis, SCSP yield reached 12.2% of the input snow crab shells, significantly higher than the chitin content (6.42%). Molecular weight distribution analysis revealed that the 5,000-10,000 Da fraction constituted the highest proportion (47.58%), followed by the 10,000-20,000 Da fraction (27.56%) (Table 3). These findings indicate that SCSP predominantly consists of low-to medium-molecular-weight peptides, a property potentially linked to its functional stability and biological activity. Amino acid composition analysis (Table 4) showed that glutamic acid (Glu) was the most abundant (5.91 g/100 g), followed by aspartic acid (Asp, 4.59 g/100 g) and lysine (Lys, 3.36 g/100 g). Essential amino acids (EAA) constituted 39.21% of the total, while non-essential amino acids (NEAA) accounted for 60.78%. The presence of both EAA and NEAA, particularly the enrichment in Glu, Asp, and Lys, suggests that SCSP may possess substantial bioactive benefits, such as promoting bone health.

**TABLE 3:** The molecular weight distribution of SCSP.

Low Limit MW	High Limit MW	Percent MW
5,000,000	2,73,380,792	0%
200,000	300,000	0%
100,000	200,000	0.94%
50,000	100,000	3.58%
30,000	50,000	6.7%
20,000	30,000	9.42%
10,000	20,000	27.56%
5,000	10,000	47.58%
4,795	5,000	4.21%

**TABLE 4:** Amino acid composition of SCSP.

Amino Acid	Content (g/100 g)
Glu	5.91
Asp	4.59
Lys	3.36
Leu	3.19
Val	2.93
Arg	2.93
Gly	2.41
Ala	2.39
IIe	2.15
Phe	2.09
Ser	1.97
Thr	1.91
Pro	1.70
Met	1.51
Tyr	1.43
His	0.98

### SCSP improves bone morphology and bone density in OVX mice

**FIGURE 1:**
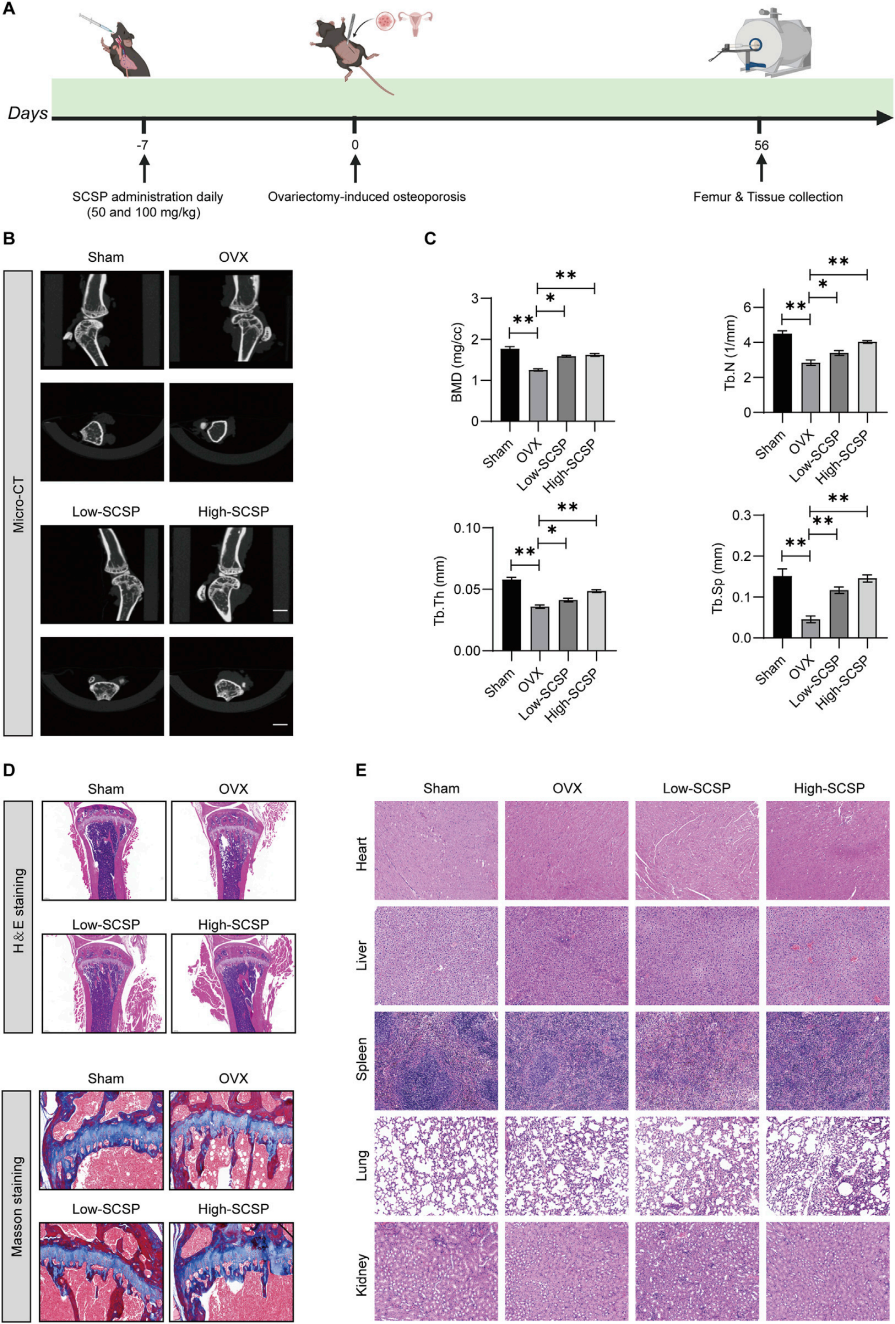
Snow crab shell-derived polypeptides (SCSP) improves bone morphology and bone density in ovariectomy (OVX) Mice. **(A)** Flow chart of animal experiment: Mice were administrated with SCSP or saline orally for 7 days, followed by bilateral ovariectomy to establish an osteoporosis model. Bone assessments were conducted on day 56 post-surgery. **(B)** Representative Micro-CT images of microstructure of proximal tibia from Sham-operated (Sham), osteoporotic model (OVX), OVX+50 mg/kg SCSP treatment (Low-SCSP), and OVX+100 mg/kg SCSP treatment (High-SCSP) groups. Scale bar, 1 mm. N = 6 mice/group. **(C)** Quantitative analysis of bone mineral density (BMD), trabecular number (Tb.N), trabecular thickness (Tb.Th), and trabecular separation (Tb.Sp) in microstructure of proximal tibia from Sham, OVX, Low-SCSP, and High-SCSP mice. Each parameter was measured 3 times, *p < 0.05, **p < 0.01, N = 6 mice/group. **(D)** Representative H&E (scale bar, 0.2 mm) and masson images (scale bar, 0.5 mm) of microstructure of proximal tibia from Sham, OVX, Low-SCSP, and High-SCSP mice. N = 6 mice/group. **(E)** Representative H&E images of heart, liver, spleen, lung and kidney tissues from Sham, OVX, Low-SCSP, and High-SCSP mice. Scale bar, 0.05 mm. N = 6 mice/group.

To investigate the therapeutic effects of SCSP on osteoporosis (OP), we utilized an ovariectomy (OVX)-induced osteoporosis mouse model (Figure 1A) and assessed bone morphology, bone mineral density (BMD), and trabecular parameters. Micro-CT scanning revealed significant trabecular degradation and reduced BMD in OVX mice compared to Sham controls, confirming osteoporotic bone loss. Treatment with SCSP at 50 mg/kg and 150 mg/kg markedly improved BMD and trabecular architecture, with the 150 mg/kg group showing bone parameters similar to those observed in the Sham group (Figure 1B). Quantitative analyses confirmed these findings, demonstrating that BMD, trabecular number (Tb.N), trabecular thickness (Tb.Th), and trabecular separation (Tb.Sp) were significantly reduced in the OVX group compared to the Sham group, while SCSP group showed notably higher as for these parameters, highlighting the protective effects of SCSP on bone quality and morphology (Figure 1C).

Histological analysis using H&E and Masson’s staining further validated these structural improvements. Bone tissue integrity and collagen fiber distribution were severely disrupted in OVX mice, whereas SCSP administration preserved these features, particularly in the 150 mg/kg group (Figure 1D). Importantly, SCSP treatment did not induce histological abnormalities in major organs, including the heart, liver, spleen, lungs, and kidneys, across all experimental groups, as evidenced by H&E staining (Figure 1E).

These findings demonstrate that SCSP alleviates OVX-induced bone loss by improving BMD, restoring trabecular architecture, and preserving bone tissue integrity, without inducing systemic toxicity.

### SCSP re-establishes the osteoblast/osteoclast balance in OVX mice

**FIGURE 2:**
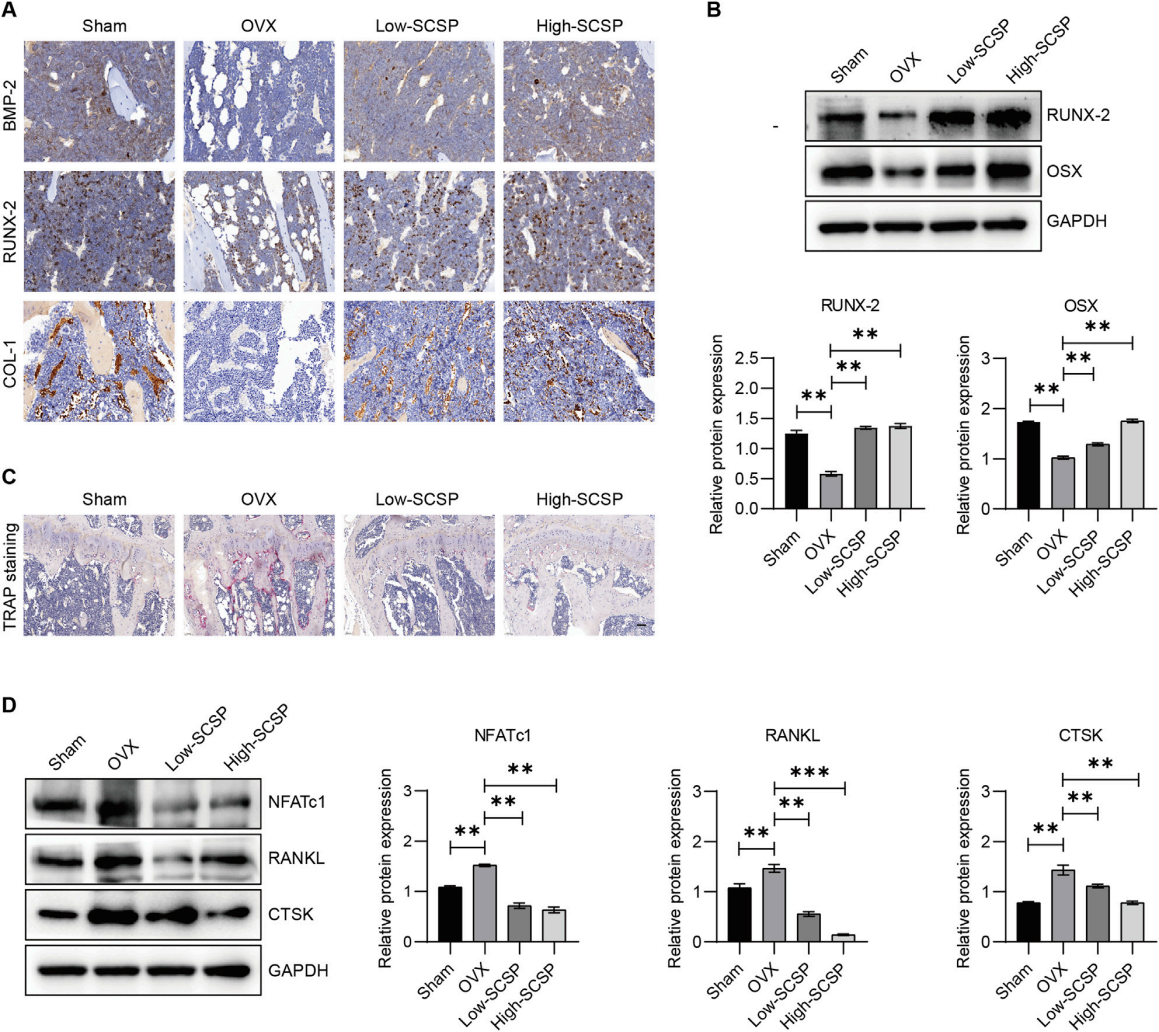
SCSP maintains the osteoblast/osteoclast balance in OVX Mice. **(A)** Representative immunohistochemistry (IHC) images of microstructure of proximal tibia stained for BMP-2, RUNX-2, and COL-1 from Sham, OVX, Low-SCSP, and High-SCSP mice. Scale bar, 0.02 mm. N = 6 mice/group. **(B)** Western blot analysis of RUNX-2 and OSX expression of microstructure of proximal tibia from Sham, OVX, Low-SCSP, and High-SCSP mice. All assays were repeated 3 times, *P < 0.05, **P < 0.01. **(C)** Representative TRAP images of microstructure of proximal tibia from Sham, OVX, Low-SCSP, and High-SCSP mice. Scale bar, 0.05 mm. N = 6 mice/group. **(D)** Western blot analysis of NFATc1, RANKL, and CTSK expression of microstructure of proximal tibia from Sham, OVX, Low-SCSP, and High-SCSP mice. All assays were repeated 3 times, *P < 0.05, **P < 0.01, ***P < 0.001.

OP is characterized by an imbalance between osteoblast-mediated bone formation and osteoclast-driven bone resorption. To assess whether SCSP modulates this balance, we analyzed markers of osteoblast and osteoclast activity. IHC staining showed significantly reduced expression of osteoblast markers (BMP-2, RUNX-2, and COL-1) in OVX mice, reflecting impaired osteoblast function (Figure 2A). SCSP administration restored these markers in a dose-dependent manner, with the 150 mg/kg group achieving levels comparable to Sham controls. Western blot analysis further confirmed increased expression of RUNX-2 and OSX in SCSP-treated groups (Figure 2B). In contrast, TRAP staining demonstrated a significant increase in osteoclast numbers in OVX mice, indicative of enhanced bone resorption (Figure 2C). SCSP treatment significantly reduced osteoclast numbers, particularly at the 150 mg/kg dose, where levels were comparable to Sham controls. Western blot analysis revealed elevated expression of osteoclast-related proteins (NFATc1, RANKL, and CTSK) in OVX mice, which was markedly reduced by SCSP treatment (Figure 2D).

With these data in hand, we examined the effects of SCSP on calcium homeostasis, which is often disrupted in OP. As expected, OVX mice exhibited reduced urinary calcium excretion (0.18 ± 0.52 mmol/L) and elevated serum phosphorus levels (2.46 ± 0.26 mmol/L). SCSP treatment increased urinary calcium and phosphorus levels above both Sham and OVX groups (Table 5). Suggesting that SCSP modulates calcium and phosphorus metabolism, potentially counteracting the metabolic disruptions induced by ovariectomy.

**TABLE 5:** Levels of serum calcium, urinary calcium, serum phosphorus and urinary phosphorus in mice ( $\bar{X}$ ± *S*).

Group	Blood Ca2+ (mmol/L)	Urine Ca2+ (mmol/L)	Blood Pi (mmol/L)	Urine Pi (mmol/L)
Sham	2.20 ± 0.16	1.22 ± 0.18	1.60 ± 0 .08	46.74 ± 1.41
OVX	2.68 ± 0.05	0.18 ± 0.52	2.46 ± 0.26	11.65 ± 0.93
Low-SCSP	2.36 ± 0.03	1.05 ± 0.22	3.92 ± 0.075	23.53 ± 2.27
High-SCSP	2.60 ± 0.13	2.75 ± 0.31	4.76 ± 0.57	49.53 ± 1.03

These results demonstrate that SCSP re-establishes the osteoblast/osteoclast balance by enhancing osteoblast activity, inhibiting osteoclast-driven bone resorption, and normalizing calcium and phosphorus metabolism.

### SCSP promotes osteogenesis and inhibit adipogenesis

**FIGURE 3:**
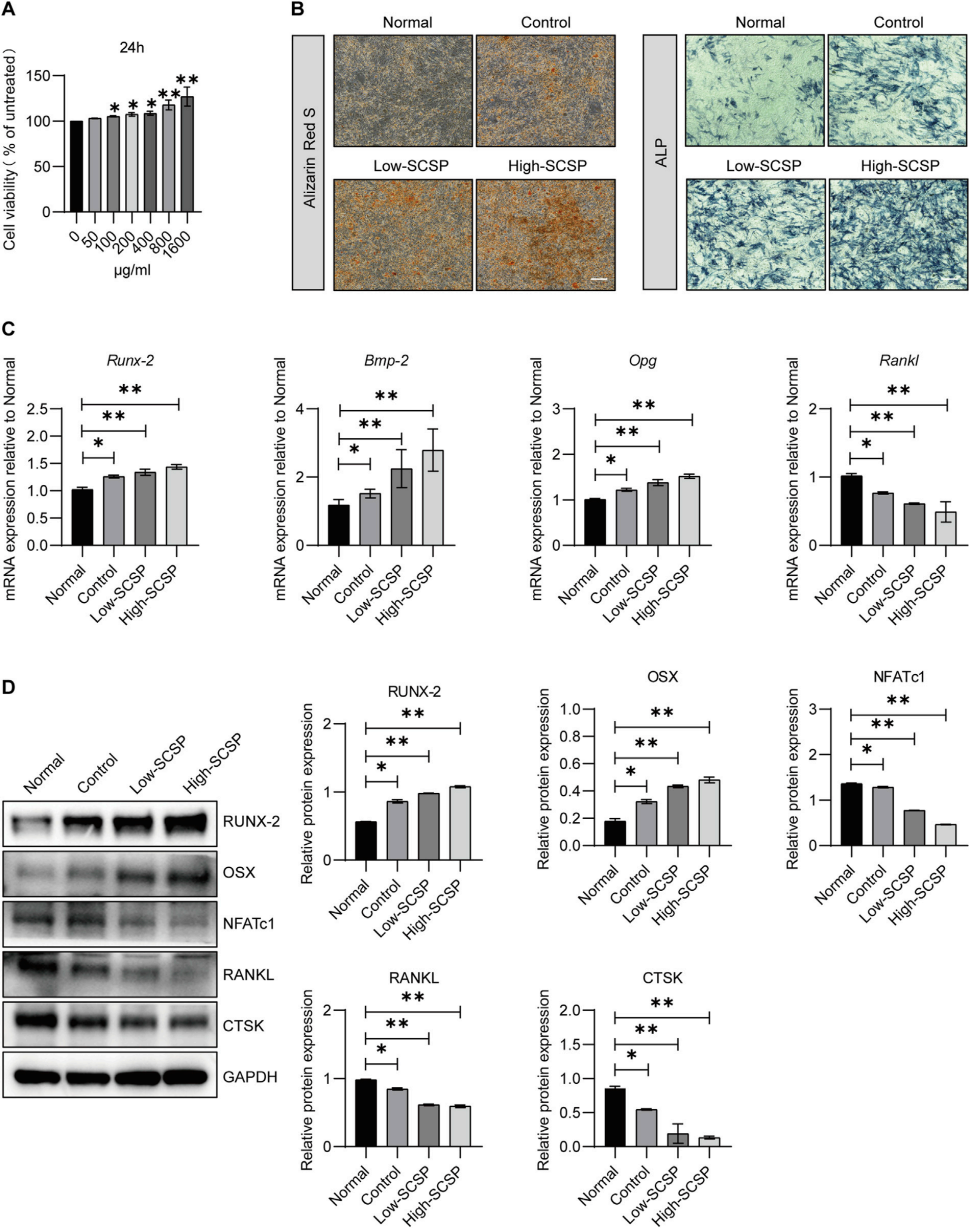
SCSP promotes osteogenic activity of MC3T3-E1 cells. **(A)** CCK8 analysis of MC3T3-E1 in the presence of SCSP ranging from 50 to 1,600 μg/mL. All assays were repeated 3 times, *P < 0.05, **P < 0.01. **(B)** Representative Alizarin Red S and ALP images of MC3T3-E1 cells treated with 100 and 200 μg/mL SCSP. All assays were repeated 3 times. Scale bar, 100 μm. **(C)** qPCR analysis of *Runx-2*, *Bmp-2*, *Opg* and *Rankl* expression from MC3T3-E1 cells treated with 100 and 200 μg/mL SCSP. All assays were repeated 3 times. *P < 0.05, **P < 0.01. **(D)** Western blot analysis of RUNX-2, OSX, NFATc1, RANKL and CTSK expression from MC3T3-E1 cells treated with 100 and 200 μg/mL SCSP. All assays were repeated 3 times. *P < 0.05, **P < 0.01.

To further elucidate the effects of SCSP on bone regeneration, we examined its influence on osteoblast proliferation, differentiation, and mineralization using MC3T3-E1 preosteoblasts cells. CCK-8 assays revealed a dose-dependent increase in osteoblast viability with SCSP treatment (Figure 3A). ARS staining indicated enhanced mineralized nodule formation and calcium deposition in SCSP-treated groups, particularly at the high dose. ALP staining confirmed enhanced early osteogenic differentiation (Figure 3B). qPCR analysis demonstrated upregulation of osteogenic genes, including *Runx-2*, *Bmp-2*, and *Opg*, and downregulation of *Rankl* expression (Figure 3C). Western blot analysis corroborated these findings, showing increased protein levels of RUNX-2 and OSX and decreased expression of NFATc1, RANKL, and CTSK (Figure 3D), suggest the role of SCSP in maintaining osteoblast/osteoclast balance.

**FIGURE 4:**
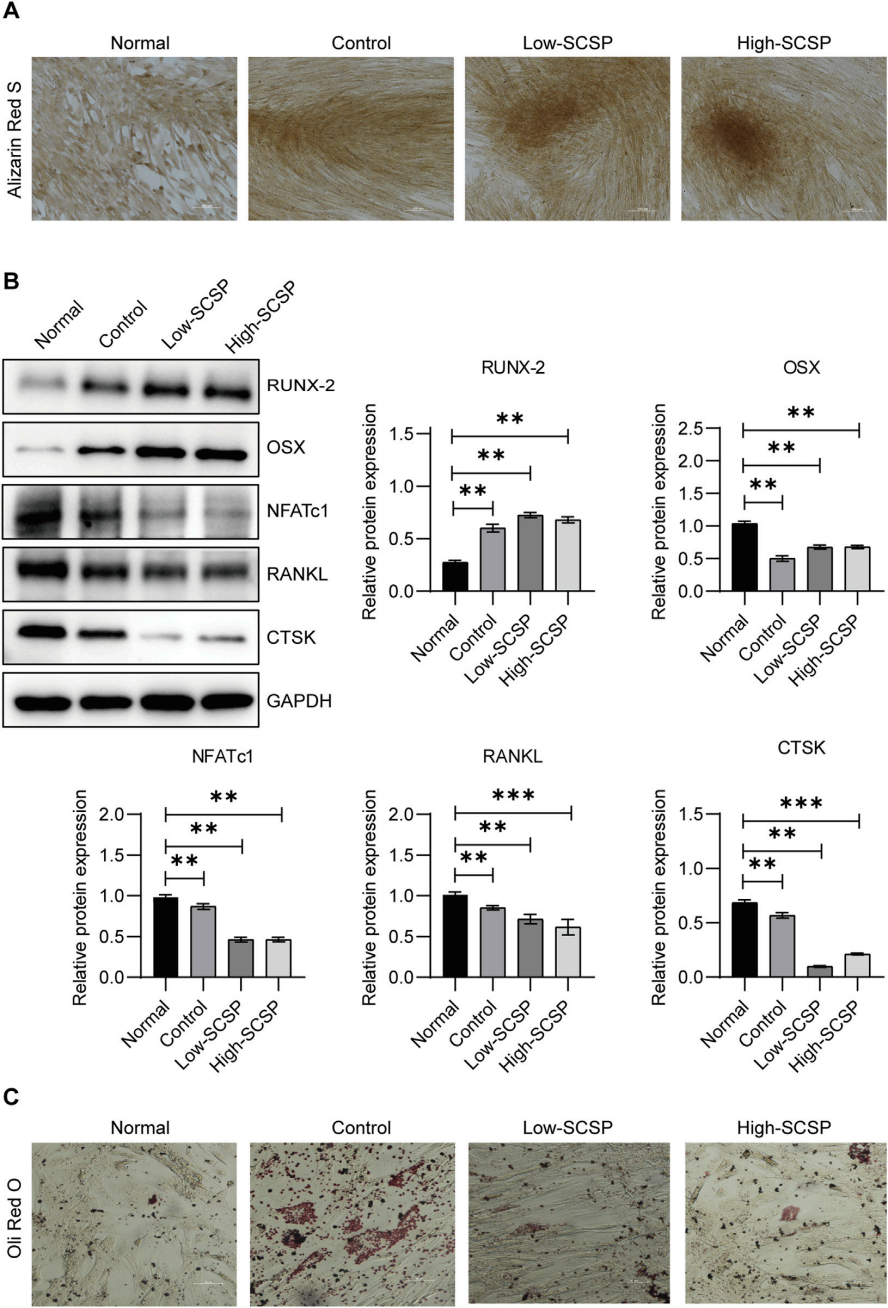
SCSP promotes osteogenic differentiation and inhibits adipogenic differentiation of mesenchymal stem cells (MSCs). **(A)** Representative Alizarin Red S images of MSCs differentiated osteoblasts treated with 100 and 200 μg/mL SCSP. Scale bar, 100 μm. All assays were repeated 3 times. **(B)** Western blot analysis of RUNX-2, OSX, NFATc1, RANKL, and CTSK expression from MSCs differentiated osteoblasts treated with 100 and 200 μg/mL SCSP. All assays were repeated 3 times. *P < 0.05, **P < 0.01, ***P < 0.001. **(C)** Representative Oil Red O images of MSCs differentiated adipocytes, Scale bar, 50 μm. All assays were repeated 3 times.

Similarly, MSCs, which can undergo both osteogenesis and adipogenesis exhibit enhanced osteogenic differentiation capacity in the presence of SCSP. ARS staining showed increased mineralized nodule formation in the SCSP-treated groups, indicating enhanced osteogenesis (Figure 4A). Western blot analysis confirmed these findings, with increased RUNX-2 and OSX expression and decreased NFATc1, RANKL, and CTSK levels in SCSP-treated groups (Figure 4B). In the contrast, SCSP treatment reduced lipid accumulation, suggesting an inhibitory effect on adipogenic differentiation (Figure 4C).

These findings highlight SCSP’s capacity to bias MSCs differentiation toward osteogenesis, promoting bone formation while inhibiting adipogenesis, and osteoclastgenesis.

### SCSP modulate cell cycle prograssion, inflammatory response, and motor protein activity in osteoblasts

**FIGURE 5:**
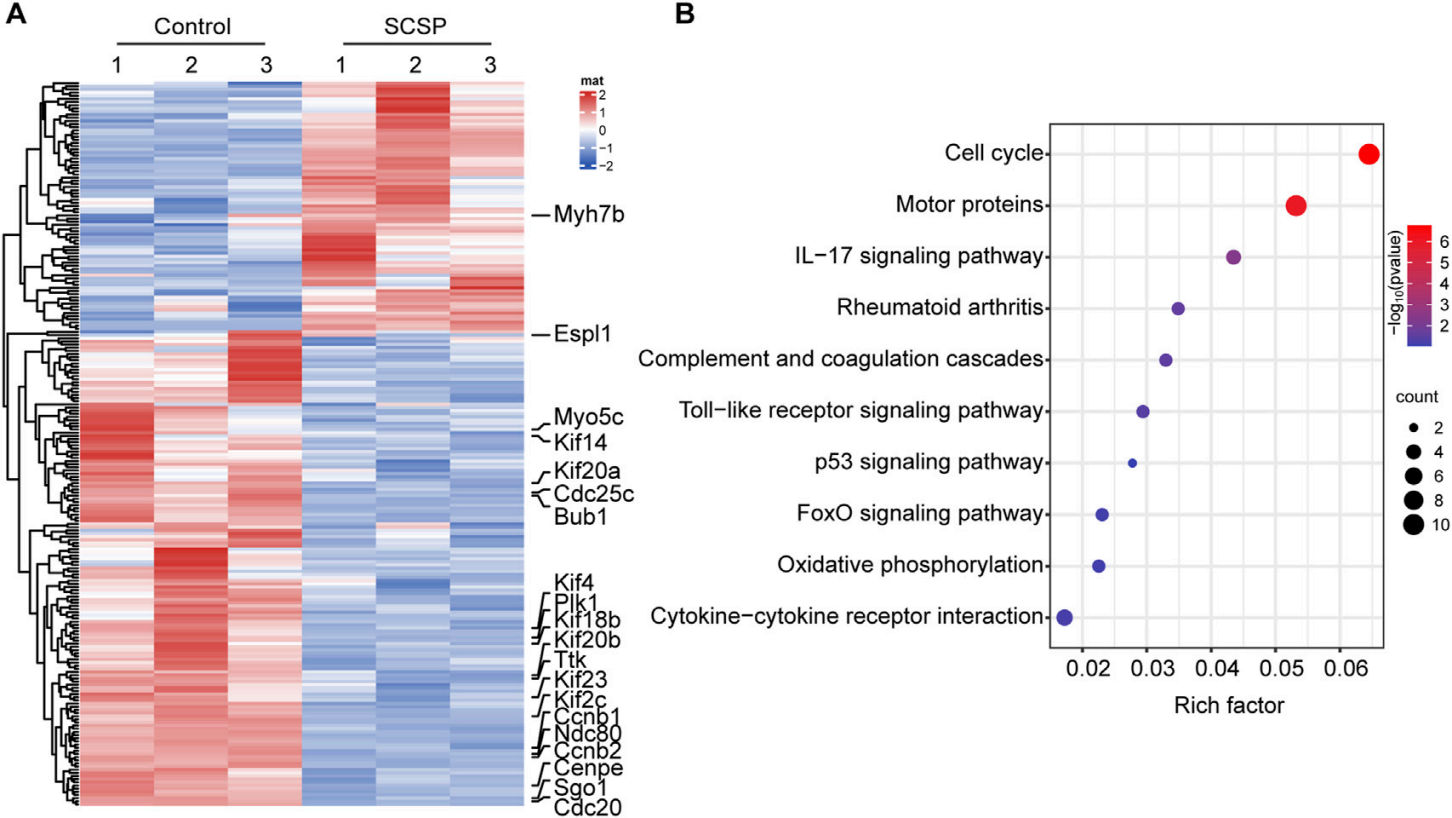
SCSP responsive genes/signaling pathways involve in osteoblasts activity. **(A)** Heatmap represents differential gene expression in RNA-Seq analysis of MC3T3-E1 cells treated with 200 μg/mL SCSP. The data from 3 biological repeat are shown as fold change greater than 2 and p values less than 0.05 were considered differentially expressed. **(B)** Analysis of KEGG pathway of differentially expressed genes from MC3T3-E1 cells treated with or without 200 μg/mL SCSP.

To obtain molecular insights into the observed above mentioned bias toward to osteogenesis, we performed RNA-Seq analysis on MC3T3-E1 differentiated osteoblast cells treated with and without 200 μg/mL of SCSP. A total of 2,410 genes were upregulated and 1,837 genes were downregulated in SCSP-treated cells compared to controls (Figure 5A). KEGG pathway enrichment analysis identified significant involvement of the Cell Cycle, inflammation, and Motor Proteins pathways (Figure 5B). Key genes involved in the Cell Cycle pathway included *Ccnb1*, *Ttk*, *Ndc80*, *Ccnb2*, *Cdc20*, *Espl1*, *Plk1*, and *Cdc25*. Among these, *Plk1*, *Ccnb2*, and *Ccnb1* are closely associated with the FoxO signaling pathway, a key regulator of osteoblast survival, oxidative stress, and bone remodeling. SCSP treatment also modulated inflammatory responses, altering expression of IL-17 signaling, Toll-like receptor signaling, and rheumatoid arthritis-related genes, including *Ccl2*, *Il17re*, *Fosl1*, *Mmp13*, *Ccl5*, *Tlr1*, *Il12b*, and *Atp6v1b1*. Notably, SCSP significantly upregulated genes associated with Motor Protein activity, including *Myo5c*, *Kif20a*, *Kif18b*, *Kif4*, *Kif23*, *Kif2c*, *Cenpe*, *Kif20b*, *Kif14*, and *Myh7b*.

These findings suggest that SCSP enhances osteoblast activity via cell cycle regulation and immunomodulation, and simultaneously modulating cytoskeletal function through Motor Proteins.

## Discussion

OP has emerged as a significant global public health issue, affecting millions worldwide (Ma C. et al., 2023). Current therapeutic strategies offer only short-term symptom relief without addressing the underlying disease mechanisms (Ma M. et al., 2023). In this study, we identify SCSP as a promising candidate for OP treatment, demonstrating its potential to effectively modulate the bone remodeling process by targeting key molecular pathways involved in osteoclastogenesis, as well as osteoblast differentiation and functions.

Our data reveal that SCSP has a molecular weight primarily within the range of 5,000-10,000 Da. Notably, smaller peptides, such as dipeptides, tripeptides, and oligopeptides, are more readily absorbed across the intestinal epithelium compared to larger proteins (Santos et al., 2012), suggesting that SCSP may undergo enzymatic degradation within the gastrointestinal tract, facilitating its absorption. The ideal molecular weight range for optimal oral bioavailability in the context of OP treatment requires further investigation to enhance SCSP absorption. Strategies such as optimizing enzymatic hydrolysis conditions or utilizing alternative enzymes to reduce peptide size could improve bioavailability, thereby enhancing its therapeutic potential (Saiwong et al., 2023; Nikoo et al., 2023; Nikoo et al., 2022). The amino acid composition of SCSP is also noteworthy. Amino acids are pivotal in mitigating age-related bone loss, enhancing bone mass, and promoting osteoblast proliferation and differentiation while concurrently suppressing osteoclast activity. Glu has been shown to be essential for osteoclast differentiation and function, as it supports the high energy demands of osteoclasts through its metabolic conversion to α-ketoglutarate, which feeds into the tricarboxylic acid cycle (Indo et al., 2013). This metabolic pathway is vital for osteoclast activity. Moreover, studies have demonstrated that depriving culture media of Glu inhibits osteoclast differentiation, indicating its critical role in osteoclastogenesis and bone resorption (Huang et al., 2021). Asp, as part of amino acid metabolism, may influence overall metabolic balance, indirectly impacting OP progression. Intriguingly, Lys, as a NEAA, promotes osteoblastogenesis by facilitating collagen crosslinking, an essential component of bone matrix formation (Goldberga et al., 2018; Jenni et al., 2016). Our chemical analysis data revealed that SCSP is abundant in Glu, Asp, and Lys, which may collectively contribute to significant bone preservation in OVX-OP models. Of interest, protein sequence and activity may vary among crustacean species. Therefore, further characterization of these crustacean shell peptides, including factors such as structure, charge, hydrophobicity, stability, binding affinity, and delivery mechanisms, is essential to achieve optimal therapeutic efficacy (Kannan et al., 2011; Zeng et al., 2021; Sharayei et al., 2021).

We show that SCSP mitigates OP progression by restoring the osteoblast/osteoclast balance, which is disrupted due to estrogen deficiency in OVX mouse model, a central trigger for RANKL/OPG dysregulation. This imbalance results in: (i) increased osteoclast activity, as estrogen normally inhibits osteoclast formation and promotes osteoclast apoptosis; (ii) reduced osteoblast activity, as estrogen stimulates osteoblast differentiation and function; and (iii) a net bone loss due to a greater rate of bone resorption than bone formation. While current treatments predominantly focus on inhibiting bone resorption to reduce bone loss, anti-resorptive agents alone cannot restore lost bone structure. In contrast, SCSP treatment addresses both osteoclast inhibition and osteoblast stimulation, making it a promising strategy for promoting bone regeneration. SCSP treatment significantly reduces the RANKL/OPG ratio, suppresses osteoclastogenesis, and enhances osteoblastic differentiation and function, as evidenced by the upregulation of osteogenic markers (RUNX2, OSX) and downregulation of osteoclast markers (NFATc1, RANKL, CTSK) in MSC and osteoblast models.

Our transcriptome analyses show that SCSP modulates pathways associated with the cell cycle, inflammatory responses, and motor protein dynamics. Cell cycle dysregulation is a hallmark of impaired bone metabolism, with senescent MSCs exhibiting reduced osteogenic potential and increased adipogenesis (Khosla et al., 2018). Senescent osteocytes and osteoclasts also secrete senescence-associated secretory phenotype factors, including pro-inflammatory cytokines, chemokines, oxidative stress mediators, and proteases, which collectively disrupt bone homeostasis (Fa et al., 2017; Collison, 2017; Paccou et al., 2019). We find that SCSP treatment inhibits Plk1 expression, supporting the differentiation and function of bone-forming cells while preventing premature senescence (Sütterlin et al., 2001; Peng et al., 2023). In addition, SCSP modulates inflammatory cascades by attenuating IL-17 signaling (Byravan et al., 2024; Peng et al., 2024), Toll-like receptor pathways (Carroll et al., 2025; He et al., 2016), and key genes implicated in inflammatory bone diseases (Lo et al., 2024), such as *Ccl2, Il17re, Fosl1, Mmp13, Ccl5, Tlr1, Il12b, and Atp6v1b1*. These anti-inflammatory effect likely contributes to the preservation of bone integrity in inflammatory OP contexts. Intriguingly, motor proteins, including myosin, dynein, and kinesin, are integral to intracellular transport (Vale, 2003), mitosis (Celestino et al., 2022), and cytoskeletal dynamics in osteoblasts and osteoclasts (Mikhajlov et al., 2025; Qiu et al., 2012; Santos-Ledo et al., 2017). SCSP’s influence on motor protein expression may enhance cellular trafficking and division, thereby supporting bone formation and remodeling processes.

Apart from the potent anti-osteoporotic effects, SCSP presents a favorable safety profile and cost-effective nature, which further strengthens its potential as a novel peptide-based therapeutic for OP. Thus, SCSP holds promise not only for the treatment of OP but also for broader applications in other skeletal diseases, providing a versatile therapeutic option for bone health management.

## Data Availability

The data generated in the present study can be found in the NCBI Sequence Read Archive database under accession number PRJNA1291493, or at the following URL: https://www.ncbi.nlm.nih.gov/sra/PRJNA1291493.
